# Air Pollution and the Microvasculature: A Cross-Sectional Assessment of In Vivo Retinal Images in the Population-Based Multi-Ethnic Study of Atherosclerosis (MESA)

**DOI:** 10.1371/journal.pmed.1000372

**Published:** 2010-11-30

**Authors:** Sara D. Adar, Ronald Klein, Barbara E. K. Klein, Adam A. Szpiro, Mary Frances Cotch, Tien Y. Wong, Marie S. O'Neill, Sandi Shrager, R. Graham Barr, David S. Siscovick, Martha L. Daviglus, Paul D. Sampson, Joel D. Kaufman

**Affiliations:** 1 Department of Epidemiology, University of Washington, Seattle, Washington, United States of America; 2 Department of Environmental and Occupational Health Sciences, University of Washington, Seattle, Washington, United States of America; 3 Department of Epidemiology, University of Michigan, Ann Arbor, Michigan, United States of America; 4 Department of Ophthalmology and Visual Sciences, University of Wisconsin, Madison, Wisconsin, United States of America; 5 Department of Biostatistics, University of Washington, Seattle, Washington, United States of America; 6 Division of Epidemiology and Clinical Applications, National Eye Institute, Bethesda, Maryland, United States of America; 7 Department of Ophthalmology, University of Melbourne, Melbourne, Victoria, Australia; 8 Singapore Eye Research Institute, National University of Singapore, Singapore; 9 Department of Environmental Health Sciences, University of Michigan, Ann Arbor, Michigan, United States of America; 10 Departments of Medicine and Epidemiology, Columbia University Medical Center, New York, New York, United States of America; 11 Department of Medicine, University of Washington, Seattle, Washington, United States of America; 12 Department of Preventive Medicine and Medicine, Northwestern University, Chicago, Illinois, United States of America; 13 Department of Statistics, University of Washington, Seattle, Washington, United States of America; Simon Fraser University, Canada

## Abstract

**Background:**

Long- and short-term exposures to air pollution, especially fine particulate matter (PM_2.5_), have been linked to cardiovascular morbidity and mortality. One hypothesized mechanism for these associations involves microvascular effects. Retinal photography provides a novel, in vivo approach to examine the association of air pollution with changes in the human microvasculature.

**Methods and Findings:**

Chronic and acute associations between residential air pollution concentrations and retinal vessel diameters, expressed as central retinal arteriolar equivalents (CRAE) and central retinal venular equivalents (CRVE), were examined using digital retinal images taken in Multi-Ethnic Study of Atherosclerosis (MESA) participants between 2002 and 2003. Study participants (46 to 87 years of age) were without clinical cardiovascular disease at the baseline examination (2000–2002). Long-term outdoor concentrations of PM_2.5_ were estimated at each participant's home for the 2 years preceding the clinical exam using a spatio-temporal model. Short-term concentrations were assigned using outdoor measurements on the day preceding the clinical exam. Residential proximity to roadways was also used as an indicator of long-term traffic exposures. All associations were examined using linear regression models adjusted for subject-specific age, sex, race/ethnicity, education, income, smoking status, alcohol use, physical activity, body mass index, family history of cardiovascular disease, diabetes status, serum cholesterol, glucose, blood pressure, emphysema, C-reactive protein, medication use, and fellow vessel diameter. Short-term associations were further controlled for weather and seasonality. Among the 4,607 participants with complete data, CRAE were found to be narrower among persons residing in regions with increased long- and short-term levels of PM_2.5_. These relationships were observed in a joint exposure model with −0.8 µm (95% confidence interval [CI] −1.1 to −0.5) and −0.4 µm (95% CI −0.8 to 0.1) decreases in CRAE per interquartile increases in long- (3 µg/m^3^) and short-term (9 µg/m^3^) PM_2.5_ levels, respectively. These reductions in CRAE are equivalent to 7- and 3-year increases in age in the same cohort. Similarly, living near a major road was also associated with a −0.7 µm decrease (95% CI −1.4 to 0.1) in CRAE. Although the chronic association with CRAE was largely influenced by differences in exposure between cities, this relationship was generally robust to control for city-level covariates and no significant differences were observed between cities. Wider CRVE were associated with living in areas of higher PM_2.5_ concentrations, but these findings were less robust and not supported by the presence of consistent acute associations with PM_2.5_.

**Conclusions:**

Residing in regions with higher air pollution concentrations and experiencing daily increases in air pollution were each associated with narrower retinal arteriolar diameters in older individuals. These findings support the hypothesis that important vascular phenomena are associated with small increases in short-term or long-term air pollution exposures, even at current exposure levels, and further corroborate reported associations between air pollution and the development and exacerbation of clinical cardiovascular disease.

*Please see later in the article for the Editors' Summary*

## Abstract 2

Sara Adar and colleagues show that residing in locations with higher air pollution concentrations and experiencing daily increases in air pollution are associated with narrower retinal arteriolar diameters in older individuals, thus providing a link between air pollution and cardiovascular disease.

## Editors' Summary

**Background:**

Cardiovascular disease (CVD)—disease that affects the heart and/or the blood vessels—is a common cause of illness and death among adults in developed countries. In the United States, for example, the leading cause of death is coronary heart disease, a CVD in which narrowing of the heart's arteries by atherosclerotic plaques (fatty deposits that build up with age) slows the blood supply to the heart and may eventually cause a heart attack (myocardial infarction). Other types of CVD include stroke (in which atherosclerotic plaques interrupt the brain's blood supply) and peripheral arterial disease (in which the blood supply to the limbs is blocked). Smoking, high blood pressure, high blood levels of cholesterol (a type of fat), having diabetes, being overweight, and being physically inactive all increase a person's risk of developing CVD. Treatments for CVD include lifestyle changes and taking drugs that lower blood pressure or blood cholesterol levels.

**Why Was This Study Done?:**

Another risk factor for CVD is exposure to long-term and/or short-term air pollution. Fine particle pollution or PM_2.5_ is particularly strongly associated with an increased risk of CVD. PM_2.5_—particulate matter 2.5 µm in diameter or 1/30th the diameter of a human hair—is mainly produced by motor vehicles, power plants, and other combustion sources. Why PM_2.5_ increases CVD risk is not clear but one possibility is that it alters the body's microvasculature (fine blood vessels known as capillaries, arterioles, and venules), thereby impairing the blood flow through the heart and brain. In this study, the researchers use noninvasive digital retinal photography to investigate whether there is an association between air pollution and changes in the human microvasculature. The retina—a light-sensitive layer at the back of the eye that converts images into electrical messages and sends them to the brain—has a dense microvasculature. Retinal photography is used to check the retinal microvasculature for signs of potentially blinding eye diseases such as diabetic retinopathy. Previous studies have found that narrower than normal retinal arterioles and wider than normal retinal venules are associated with CVD.

**What Did the Researchers Do and Find?:**

The researchers used digital retinal photography to measure the diameters of retinal blood vessels in the participants of the Multi-Ethnic Study of Atherosclerosis (MESA). This study is investigating CVD progression in people aged 45–84 years of various ethnic backgrounds who had no CVD symptoms when they enrolled in the study in 2000–2002. The researchers modeled the long-term outdoor concentration of PM_2.5_ at each participant's house for the 2-year period preceding the retinal examination (which was done between 2002 and 2003) using data on PM_2.5_ levels collected by regulatory monitoring stations as well as study-specific air samples collected outside of the homes and in the communities of study participants. Outdoor PM_2.5_ measurements taken the day before the examination provided short-term PM_2.5_ levels. Among the 4,607 MESA participants who had complete data, retinal arteriolar diameters were narrowed among those who lived in regions with increased long- and short-term PM_2.5_ levels. Specifically, an increase in long-term PM_2.5_ concentrations of 3 µg/m^3^ was associated with a 0.8 µm decrease in arteriolar diameter, a reduction equivalent to that seen for a 7-year increase in age in this group of people. Living near a major road, another indicator of long-term exposure to PM_2.5_ pollution, was also associated with narrowed arterioles. Finally, increased retinal venular diameters were weakly associated with long-term high PM_2.5_ concentrations.

**What Do These Findings Mean?:**

These findings indicate that living in areas with long-term air pollution or being exposed to short-term air pollution is associated with narrowing of the retinal arterioles in older individuals. They also show that widening of retinal venules is associated with long-term (but not short-term) PM_2.5_ pollution. Together, these findings support the hypothesis that long- and short-term air pollution increases CVD risk through effects on the microvasculature. However, they do not prove that PM_2.5_ is the constituent of air pollution that drives microvascular changes—these findings could reflect the toxicity of another pollutant or the pollution mixture as a whole. Importantly, these findings show that microvascular changes can occur at the PM_2.5_ levels that commonly occur in developed countries, which are well below those seen in developing countries. Worryingly, they also suggest that the deleterious cardiovascular effects of air pollution could occur at levels below existing regulatory standards.

**Additional Information:**

Please access these Web sites via the online version of this summary at http://dx.doi.org/ 10.1371/journal.pmed.1000372.

- The American Heart Association provides information for patients and caregivers on all aspects of cardiovascular disease (in several languages), including information on air pollution, heart disease, and stroke
- The US Centers for Disease Control and Prevention has information on heart disease and on stroke
- Information is available from the British Heart Foundation on cardiovascular disease
- The UK National Health Service Choices website provides information for patients and caregivers about cardiovascular disease
- MedlinePlus provides links to other sources of information on heart disease and on vascular disease (in English and Spanish)
- The AIRNow site provides information about US air quality and about air pollution and health
- The Air Quality Archive has up-to-date information about air pollution in the UK and information about the health effects of air pollution
- The US Environmental Protection Agency has information on PM2.5
- The following Web sites contain information available on the MESA and MESA Air studies

## Introduction

Long- and short-term exposures to ambient and traffic-related air pollution, especially fine particulate matter (particulate matter less than 2.5 µm in aerodynamic diameter, or PM_2.5_), have been linked to higher rates of cardiovascular morbidity and mortality [1]–[5]. It has been hypothesized that impacts on microvascular function may play a role in these associations [6]. Toxicological studies support this hypothesis as they have demonstrated that PM_2.5_ can impair microvascular endothelium-dependent dilation [7]. One human study also demonstrated air pollution and exercise-induced ischemia in a pattern that was more consistent with impaired myocardial microvascular flow than altered larger epicardial vessel coronary circulation [8]. Other human studies have shown links between air pollution and impaired vasodilatation or enhanced vasoconstriction in the forearm [9]–[12], but none have directly explored associations with the microvasculature.

Retinal photography provides a noninvasive, in vivo, method for characterizing the human microvasculature since retinal vessels are 60–300 µm in diameter. Several studies have found that narrower arteriolar diameters and wider venular diameters, as measured by retinal photography, are each associated with increased risk of myocardial infarction, stroke, and cardiovascular mortality, independent of other traditional risk factors [13]–[19]. Furthermore, recent data from the Multi-Ethnic Study of Atherosclerosis (MESA) have linked narrower retinal arteriolar diameters with measures of subclinical cardiovascular disease including increased left ventricular mass, reduced aortic distensibility, and reduced large artery compliance [20]–[22]. These findings were independent of other cardiovascular risk factors, including blood pressure, diabetes, blood cholesterol level, family history of cardiovascular disease, body mass index (BMI), glucose level, and C-reactive protein, which have also been associated with retinal vessel diameter in the same cohort [23]. It is unknown, however, if exposures to ambient and traffic-related air pollution are related to adverse retinal microvascular changes

Our primary aim was to investigate cross-sectional associations between long- and short-term air pollution concentrations and microvascular characteristics using arteriolar vessel diameter as measured by retinal photography in MESA, a well-characterized cohort with individual-level predictions of residential pollutant concentrations. We hypothesized that higher air pollution levels would be associated with narrowed retinal arteriole diameters. We also hypothesized that greater air pollution concentrations would be associated with widened retinal venular diameters.

## Methods

### Study Population

MESA is a prospective cohort study designed to investigate the progression of subclinical and clinical cardiovascular disease [24]. The cohort is comprised of 6,814 white, African American, Hispanic, and Asian (of Chinese decent) men and women recruited in six American communities (Baltimore, MD; Chicago, IL; Forsyth County, NC; Los Angeles County, CA; Northern Manhattan, NY; and St. Paul, MN). Participants were aged 45 to 84 y and free of clinical cardiovascular disease at their baseline exam (July 2000 to August 2002). This study met with the guidelines of the Declaration of Helsinki. Institutional review board approval was granted at each study site, and written informed consent was obtained from all participants. Only participants with complete retinal, exposure, and covariate information were included in this analysis.

### Retinal Photography and Grading

Retinal photography was performed at each field center using standardized methods during the second MESA examination, which took place between August 2002 and January 2004. Of the 6,233 participants that returned for this follow-up exam, 6,176 individuals had retinal photographs taken. For each participant, the optic disc and macula of both eyes were photographed in a darkened room using a 45° 6.3-megapixel digital nonmydriatic camera (Canon), and methods described in detail by Klein and colleagues [25]. Trained graders who were masked to all participant characteristics reviewed all images at the University of Wisconsin Ocular Epidemiology Reading Center in Madison using previously reported protocols [26]–[28]. Briefly, retinal vessel diameters were measured using a computer-based program and summarized as central retinal arteriolar and venular equivalents (CRAE and CRVE, respectively). These equivalents represent a summary of vessel diameters within an area equal to 0.5–1 disc diameters from the optic disc margin. Vessel diameters for the right eye were selected for all analyses. Images from the left eye were used only if retinal vessel diameters could not be graded in the right eye. The reproducibility of the vessel diameter measurements in MESA were good, with intragrader and intergrader correlation coefficients of 0.78 to 0.99 [26].

### Participant Characteristics

Detailed data regarding participant health were collected during the first and second MESA clinical exams, including anthropometry as well as serum levels of high-density lipoprotein (HDL) and low-density lipoprotein (LDL) cholesterol, glucose, homocysteine, and inflammatory markers [24]. Information regarding participant demographics, medical history, and medication use was also obtained at these exams through technician-administered questionnaires. Residential addresses provided at the exams were assigned geographic coordinates using ArcGIS software, version 9.1 (ESRI), on the basis of the Dynamap/2000 street network (Tele Atlas).

City-wide covariates were also collected for each metropolitan statistical area from the US Census 2000 (http://www.census.gov/main/www/cen2000.html) and used in sensitivity analyses. Variables included information about the general population's age, race/ethnicity, educational attainment, nativity, unemployment, income, and poverty status. Consistent with the reanalysis of the Harvard Six Cities and the American Cancer Society Studies [29], we also explored confounding by city-average elevation, maximum temperature, and the variation in temperature as expressed by the standard deviation of daily readings.

### Exposure Assignment

Estimates of long-term ambient PM_2.5_ concentrations were computed for each participant using a hierarchical spatio-temporal model fit with a pragmatic estimation procedure described elsewhere [30],[31]. The data utilized to derive these predictions were 2-wk average concentrations of PM_2.5_ collected over the course of several years by regulatory monitoring stations from the US Environmental Protection Agency's Air Quality System (AQS) and supplemental monitoring stations specific to this project [32]. Briefly, the hierarchical model decomposed the space-time field of concentrations into three pieces: (i) spatially varying long-term averages, (ii) spatially varying seasonal and long-term trends, and (iii) spatially correlated but temporally independent residuals. Model estimation and prediction were carried out using a multistep procedure that incorporated a large suite of spatial covariates such as proximity to major roadways and local land use to predict average PM_2.5_ concentrations at each subject's home location over the 2 y preceding the participant's exam date. Model selection and validation were carried out using cross-validation with city-specific cross-validated root mean square errors for predicting long-term average concentrations at Multi-Ethnic Study of Atherosclerosis and and Air Pollution (MESA Air) monitoring locations between 0.34 and 0.94 µg/m^3^ [30].

Since our hierarchical model is currently only resolved to the 2-wk time scale, we did not apply this approach for our short-term analysis. Rather, city-wide average concentrations from AQS monitoring stations with complete time series during the period of interest were used to estimate daily concentrations of PM_2.5_. This approach is consistent with the majority of acute air pollution epidemiology research and is justified by the fact that daily PM_2.5_ concentrations are typically highly correlated across different locations within a region over time. On the basis of our hypothesis that acute impacts on the microvasculature would be rapid, we assigned concentrations for each participant for the day of their exam, the preceding day, and the average of the preceding 3 d. A priori, we selected concentrations on the day preceding the exam for our primary analyses, although we evaluated the other two time periods in sensitivity analyses.

In other sensitivity analyses, we explored the relationship between both outcomes and long-term average concentrations of PM_2.5_ measured at the nearest AQS monitor as a more traditional indicator of pollution. Additionally, we evaluated the impact of living near a major roadway as defined by living within 100 m of an interstate or US highway (Census Feature Class Code A1 or A2) or within 50 m of a state or county highway (CFCC A3). These distances were calculated in ArcGIS 9.1 on the basis of the TeleAtlas Dynamap 2000 road network.

### Data Analysis

Linear regression modeling was performed in R version 2.10.1 (The R Project) in order to examine cross-sectional associations between air pollution and retinal vessel diameters. Long-and short-term associations with air pollution concentrations were modeled independently and jointly. For both CRAE and CRVE, we performed our modeling in a phased approach and explored confounding by age, sex, race/ethnicity (white, African American, Chinese American, Mexican Hispanic, Dominican Hispanic, Puerto Rican Hispanic, and other Hispanic), and education (<high school, high school or equivalent, associate, bachelor, or technical degree, and graduate degree). We also considered lifestyle factors including smoking status (never, former, and current), alcohol consumption (user, nonuser), and physical activity in metabolic equivalent (MET)-minutes/week. In addition, confounding by BMI, waist to hip ratio, family history of cardiovascular disease (defined by a history of heart attack, stroke, or noninjury amputation among a parent, sibling, or child), history of diabetes mellitus (defined by the 2003 American Diabetes Association fasting criteria algorithm [33]), systolic and diastolic blood pressure, emphysema, as well as serum glucose, HDL and LDL cholesterol, homocysteine, and inflammatory markers (C-reactive protein and fibrinogen) levels was explored. On the basis of past research, which indicates that anatomical variation and measurement error in retinal caliber can lead to spurious associations [34]–[36], we also explored confounding between CRAE and CRVE. By controlling for the fellow vessel type in our models, we are further able to address the scientific question of how air pollution is associated with vessel caliber relative to the fellow vessel. Although examined, income (<US$20,000, US$20,000 to <US$50,000, US$50,000 to <US$75,000, ≥US$75,000), secondhand smoke exposures, and medication use (antihypertensives, lipid-lowering medications, aspirin, cox-2 inhibitors, erectile dysfunction medications, beta agonists, sympathomimetics, verapamil, and vasodilators) were ultimately not included in our main models because there were more missing data for these parameters and sensitivity analyses demonstrated little impact on our results. While not of primary interest, we reported effect estimates for blood pressure and age on vessel diameters from the fully adjusted models for comparison purposes.

Linearity of all continuous variables was checked using LOESS smoothing fits. For CRAE, all variables were modeled as linear except for systolic blood pressure, which was modeled as a piecewise linear spline with a knot at 135 mmHg and BMI, which was modeled as a quadratic. For CRVE, only HDL cholesterol was modeled with a nonlinear function expressed as a piecewise linear spline with a knot at 100 mg/dl. For both outcomes, homocysteine levels were modeled using a log-transformation to reduce the impact of highly skewed values on the results. For our acute effect analyses, we included categorical variables for city and city-specific day of week effects as well as spline fits for city-specific trends in calendar day (12 degrees of freedom [df]/year), temperature (6 df), and dew point (6 df) using B-splines to control for confounding by seasonality and meteorology on the day of the clinical exam.

To explore the importance of site on our models, we first tested for statistically significant interactions by city. We then decomposed our PM_2.5_ concentrations into between- and within-city exposure contrasts to examine the contribution of each to any observed associations and investigated results stratified by city. Finally, we explored the possibility of confounding of our overall associations by individual city-level factors. We further investigated differential susceptibility by including interaction terms for age, race/ethnicity, gender, obesity, diabetes, hypertension, use of specific classes of hypertensive medications, and use of anti-inflammatory medications.

## Results

With retinal data missing for 283 participants, address or exposure data missing for 1,104 persons, and covariate data missing for 243 persons, 4,607 individuals were examined in this analysis. Descriptive statistics are presented in Table 1 for individuals with complete information as well as those individuals excluded from analysis. Included participants were 53% women among four race/ethnicities (40% white, 26% African American, 12% Chinese American, and 22% Hispanic). Of the Hispanics in the population, 52%, 14%, 14%, and 35% were of Mexican, Dominican, Puerto Rican, and other descent, respectively. With a mean age of 63 y, 44% of the population had hypertension, 13% had diabetes (an additional 15% had impaired glucose), and 58% had a family history of cardiovascular disease. Excluded individuals were qualitatively similar to the main cohort although they were slightly older, of lower socioeconomic position, and had higher prevalence of hypertension and diabetes.

**Table 1:** Demographic and health characteristics of the MESA participants in exam 2.

Characteristics	All Participants		Participants with Complete Data		Excluded Participants	
	*n*	Mean (SD) or Percent	*n*	Mean (SD) or Percent	*n*	Mean (SD) or Percent
**Personal Characteristics**						
Age (y)	6,233	64 (10)	4,607	63 (10)^a^	1,626	66 (11)^a^
Female (%)	3,241	52	2,439	53	801	49
Race/ethnicity (%)						
White	2,493	40	1,853	40	611	38
African American	1,683	27	1,212	26	479	29
Chinese	748	12	537	12	191	12
Hispanic	1,371	22	1,005	22	345	21
Annual income (thousands US$)				^a^		^a^
<20	1,377	23	941	21[Table-fn nt101]	451	29[Table-fn nt101]
20 to <50	2,276	38	1,673	38	580	38
50 to <75	958	16	733	16	214	14
>75	1,377	23	1,107	25	290	19
Education (%)						^a^
Less than high school	994	16	714	15	330	21[Table-fn nt101]
High school	2,175	35	1,573	34	564	35
Higher education	1,865	30	1,429	31	445	28
Advanced degree	1,181	19	891	19	269	17
Smoking status (%)						
Never	2,847	46	2,149	47	708	45
Former	2,661	43	1,960	43	677	43
Current	681	11	498	11	197	12
Current alcohol user (%)	3,172	51	2,405	52	774	48^a^
Exercise (MET min/wk)	6,223	1,395 (2,106)	4,607	1450 (2200)	1,615	1,230 (1,800)^a^
**General health characteristics**						
Waist-hip ratio (%)	6,230	93 (8)	4,607	93 (8)	1,623	94 (8)^a^
Systolic BP (mm Hg)	6,230	124 (21)	4,607	123 (20)^a^	1,623	128 (22)^a^
Diastolic BP (mm Hg)	6,230	70 (10)	4,607	70 (10)	1,623	71 (10)
HDL (mg/dl)	6,183	52 (15)	4,607	52 (15)	1,576	51 (15)
LDL (mg/dl)	6,114	114 (32)	4,607	113 (32)	1,507	114 (33)
Fibrinogen (mg/dl)	6,194	345 (73)	4,607	344 (73)	1,587	350 (72)^a^
CRP (mgl)	6,191	3.7 (5.4)	4,607	3.6 (5.3)	1,584	3.8 (5.8)
Glucose (mg/dl)	6,184	100 (30)	4,607	99 (28)^a^	1,577	104 (35)^a^
Homocysteine (µmol/l)	6,217	9.3 (3.8)	4,607	9.1 (3.5)^a^	1,610	9.7 (4.1)^a^
Family history of CVD (%)	3,625	58	2,681	58	944	58
Hypertension (%)[Table-fn nt101]	2,838	46	2,008	44^a^	820	51^a^
Diabetes (%)[Table-fn nt101]	928	15	622	13^a^	294	19^a^
Antihypertensive medication (%)[Table-fn nt101]	2,508	42	1,808	41	703	45^a^
Lipid lowering medication (%)	1,313	22	987	22	354	23

a Statistically significant differences from the main population at the 95% confidence level.

BP, blood pressure; CRP, C-reactive protein; CVD, cardiovascular disease; MET, metabolic equivalent.

The mean CRAE in the population was 144 µm and the mean CRVE was 214 µm (Table 2). Long-term concentrations of outdoor PM_2.5_ were similar between our spatio-temporal model predictions and the closest EPA-monitoring station with means of 16 µg/m^3^ across all participants and standard deviations of 3 µg/m^3^. As anticipated, short-term concentrations were more variable than long-term averages (standard deviations [SDs] of 9 µg/m^3^ for the shortest time frames) and low correlations were found between our short- and long-term average concentrations (0.25 in crude analyses and 0 after adjustment for site). Approximately 30% of the population was living near a major roadway.

**Table 2:** Distribution of retinal outcomes and long-term (previous 2 y) and short-term (previous days) exposure estimates among 4,607 participants with complete data.

Variable	Mean	SD	Percentile				
			Minimum	25th	50th	75th	Maximum
**Outcome measures**							
CRAE (µm)	144	14	83	135	144	153	219
CRVE (µm)	214	22	124	199	214	228	324
**Long-term exposure estimates**							
Personal PM_2.5_ predictions (µg/m^3^)	16.3	3.4	9.6	14.6	15.9	17.2	26.3
Nearest monitor PM_2.5_ (µg/m^3^)	16.1	3.3	9.6	12.8	15.9	17.3	25.4
**Short-term exposure estimates**							
Concurrent day PM_2.5_ (µg/m^3^)	15.8	9.3	2.1	9.1	13.7	19.7	69.3
Previous day PM_2.5_ (µg/m^3^)	15.4	9.1	1.4	9.0	13.5	19.6	97.6
Previous 3-d PM_2.5_ (µg/m^3^)	15.2	7.4	3.6	10.0	13.4	18.3	58.6

### Retinal Arteriolar Diameters

In multivariate regression models, CRAE was negatively associated with long-term average modeled PM_2.5_ levels with a greater magnitude of association seen with increasing control for potentially confounding variables (Table 3). The strongest effect estimate was found in the model fully adjusted for all personal, health, lifestyle characteristics, and CRVE. Similar results were found in a model that included concentrations of PM_2.5_ on the day preceding the health exam. In this final joint model, an interquartile increase of 3 µg/m^3^ in our modeled long-term PM_2.5_ concentrations was associated with a −0.8 µm (95% confidence interval [CI] −1.1 to −0.5) decrease in CRAE. This association was quantitatively similar to a 7-y increase in age or a 3-mm Hg increase in diastolic blood pressure in our predictive models for CRAE.

**Table 3:** Associations between retinal diameters and long- (previous 2 y) and short-term (previous day) exposures to fine particulate air pollution.

Model	Retinal Arteriolar Diameter (CRAE)		Retinal Venular Diameter (CRVE)	
	Long-Term Effects	Short-Term Effects	Long-Term Effects	Short-Term Effects
1	−0.5 (−0.9 to −0.1)	−0.4 (−0.8 to −0.04)	0.3 (−0.3 to 0.9)	−0.1 (−0.7 to 0.5)
2	−0.5 (−0.9 to −0.1)	−0.6 (−1.0 to −0.2)	0.5 (−0.04 to 1.1)	−0.1 (−0.7 to 0.5)
3	−0.8 (−1.1 to −0.4)	−0.5 (−1.0 to 0.03)	0.3 (−0.2 to 0.9)	−0.1 (−0.9 to 0.7)
4	−0.9 (−1.2 to −0.6)	−0.4 (−0.8 to 0.1)	0.9 (0.4 to –1.4)	0.4 (−0.3 to 1.1)
Joint	−0.8 (−1.1 to −0.5)	−0.4 (−0.8 to 0.1)	0.9 (0.4 to –1.4)	0.4 (−0.3 to 1.1)

All associations reported as µm per interquartile range of 3 µg/m^3^ (for long term) and 9 µg/m^3^ (for short term). For both long- and short-term associations, model 1 controlled for age, sex, and race/ethnicity. In our long-term analyses, model 2 also included control for BMI, waist-to-hip ratio, income, education, smoking history, alcohol use, and family history of cardiovascular disease. Model 3 of our long-term analysis and Model 2 of our short-term analysis added control for LDL, HDL, blood pressure, diabetes, glucose, physical activity, emphysema, CRP, fibrinogen, and homocysteine. In our short-term analyses, model 2 controlled for all variables in model 3 of our long-term analysis while model 3 also included city-specific trends for day of week, time, temperature, and relative humidity. Model 4 added control for the fellow vessel diameter (e.g., CRAE or CRVE) to each models 3, and the joint model included long- and short-term concentrations simultaneously with control for covariates listed in the two respective models 4.

**Figure 1:**
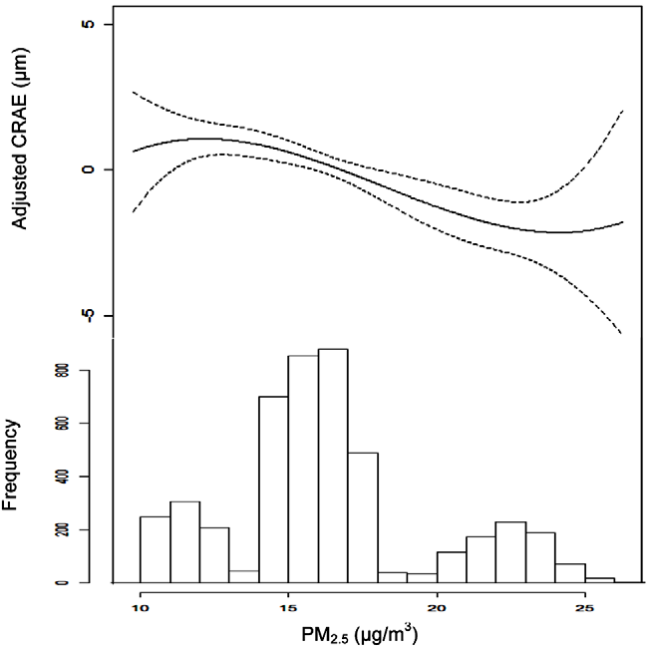
Associations between retinal arteriolar diameter (CRAE) and modeled long-term PM_2.5_ concentrations after control for covariates. Note: CRAE values represent residuals from full joint model (i.e., model controlled for age, sex, race/ethnicity, BMI, waist-to-hip ratio, income, education, smoking history, alcohol use, family history of cardiovascular disease, LDL, HDL, blood pressure, diabetes, glucose, physical activity, emphysema, CRP, fibrinogen, homocysteine, CRVE, and previous day PM_2.5_ concentration). Data are plotted as a cubic polynomial with 3 df.

**Figure 2:**
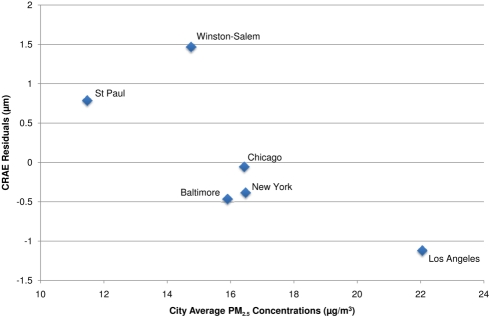
City-wide associations between CRAE and modeled long-term PM_2.5_ concentrations after control for covariates. Notes: CRAE values presented as the residuals from a model controlled for age, sex, race/ethnicity, BMI, waist-to-hip ratio, income, education, smoking history, alcohol use, family history of cardiovascular disease, LDL, HDL, blood pressure, diabetes, glucose, physical activity, emphysema, CRP, fibrinogen, homocysteine, CRVE, and previous day PM_2.5_ concentration.

Figure 1 illustrates the chronic relationship between CRAE and the underlying trimodal distribution of long-term PM_2.5_ concentrations in the cohort with clusters of individuals living in St. Paul and Winston Salem (the lowest polluted cities), New York, Chicago, and Baltimore (the three modestly polluted cities), and Los Angeles (the most polluted city). Given the larger variation in long-term average concentrations between cities (SD = 3.3 µg/m^3^) as compared to within-cities (SD = 1.1 µg/m^3^), our overall results were driven by differences in air pollution levels between cities. This finding is illustrated by Figure 2, which shows the largest city-wide average retinal diameters among participants in the two least polluted cities and the smallest city-wide average diameters among persons in the most polluted city, after control for all confounders in a model including short-term concentrations. Partitioning the PM_2.5_ variation into city-wide means and deviations from that mean, we found that between-city differences in concentration were associated with a −0.9 µm (95% CI −1.2 to −0.5) decrease in CRAE per 3 µg/m^3^. Controlling for site using a random intercepts and random slopes model, we found that a 3 µg/m^3^ increase in PM_2.5_ was associated with a −0.7 µm (95% CI −1.2 to −0.1) decrease in CRAE. Controlling for site as a fixed effect resulted in a decrease of −0.4 µm (95% CI −1.3 to 0.5) per 3 µg/m^3^.

In spite of weaker and less precise estimates within city, we found no evidence of statistically significant differences in association by city (*p*-value for interaction = 0.6). In addition, our overall results (which include between and within-city variability in PM_2.5_) were robust to exclusion of any of the six cities, with the weakest association found when excluding Baltimore, New York, or Winston-Salem (−0.8 µm [95% CI −1.1 to −0.4] decrease per 3 µg/m^3^) and the strongest association found when excluding St. Paul (−1.1 µm [95% CI −1.6 to −0.7] decrease per 3 µg/m^3^). Furthermore, control for a variety of city-wide covariates including age, race/ethnicity, socioeconomic status, weather, and elevation did not qualitatively change our findings (unpublished data).We also found consistency between our modeled estimates of long-term average PM_2.5_ concentrations and the long-term average PM_2.5_ concentrations directly measured at the EPA monitoring station nearest the participants' residences, which also had negative, although generally smaller, associations with CRAE. Negative associations with larger CIs were also found for residential proximity to major roadways. In the fully adjusted, joint models, a 3-µg/m^3^ increase in PM_2.5_ measured at the nearest monitor was associated with a −0.5 µm decrease (95% CI −0.8 to −0.1) in CRAE and living close to a major roadway was associated with a −0.7 µm decrease (95% CI −1.4 to 0.1) in CRAE.

For short-term concentrations of PM_2.5_, we found that with CRAE, a 9-µg/m^3^ increase in exposure the day prior to the exam (the interquartile difference) was associated with a −0.4-µm decrease (95% CI −0.8 to 0.1, *p* = 0.09) in CRAE in the fully adjusted model, which included chronic air pollution exposures (Table 3). These associations were insensitive to adjustment for long-term concentrations and city, although they were slightly weakened by control for meteorology and other vessel diameter. Some sensitivity to exposure window was also found with the strongest point estimates of decreased diameter generally observed for our *a priori* hypothesized exposure period of the previous day. Decreases of −0.2 µm (95% CI −0.6 to 0.3) and −0.1 µm (95% CI −0.5 to 0.3) were seen for a 9 µg/m^3^ increase in the current day and 7 µg/m^3^ increase in the previous 3 d concentrations, respectively.

Only gender appeared to significantly modify the association between long-term exposures to air pollution and CRAE, with men demonstrating a larger reduction in retinal diameter (−1.3 µm [95% CI −1.7 to −0.8]) than women (−0.4 µm [95% CI −0.8 to 0.1]) for the same difference (3 µg/m^3^) in PM_2.5_ concentrations. These findings were not found in the short-term analysis, and no other factors were found to significantly modify the associations between air pollution and CRAE, including the presence of diabetes and race/ethnicity.

### Retinal Venular Diameters

**Figure 3:**
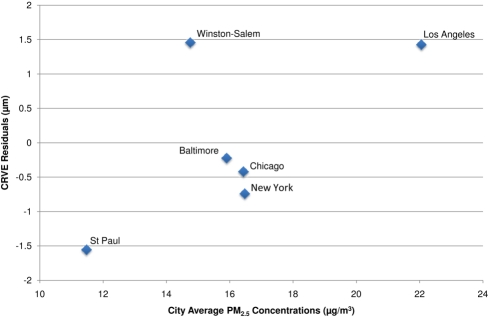
City-wide associations between CRVE and modeled long-term PM_2.5_ concentrations after control for covariates. Notes: CRVE values presented as the residuals from a model controlled for age, sex, race/ethnicity, BMI, waist-to-hip ratio, income, education, smoking history, alcohol use, family history of cardiovascular disease, LDL, HDL, blood pressure, diabetes, glucose, physical activity, emphysema, CRP, fibrinogen, homocysteine, CRAE, and previous day PM_2.5_ concentration.

As hypothesized, long-term PM_2.5_ concentrations were associated with wider venular diameters (larger CRVE) in our multivariate models (Table 3). The strongest association was found in the fully adjusted model, including control for CRAE, with similar results for the model that included concentrations of PM_2.5_ on the day preceding the health exam. An interquartile increase of approximately 3 µg/m^3^ in long-term average PM_2.5_ was associated with a 0.9 µm (95% CI 0.4–1.4 µm) increase in CRVE. Partitioning the PM_2.5_ variability into a city mean and deviations from that mean, we found that between-city differences were more strongly and significantly associated with CRVE (1.0 µm [95% CI 0.4–1.5] per 3 µg/m^3^) than were pooled within-city differences (0.5 µm [95% CI −0.8 to 1.9] per 3 µg/m^3^). In fact, sensitivity analyses demonstrated that these results were driven by between city differences on the basis of the cities with the highest (Los Angeles) and lowest (St. Paul) concentrations (Figure 3). Exclusion of either of these cities resulted in a 20%–30% reduction in the effect estimate and a loss of statistical significance. Like for CRAE, analyses of individual cities demonstrated highly variable results although St. Paul was found to have a statistically significant association with CRVE with a 7.0 µm (95% CI 1.5–12.6) increase predicted per 3 µg/m^3^ in PM_2.5_ (results not shown).

Associations derived using modeled concentrations and concentrations measured directly at the nearest EPA monitor were similar to our spatio-temporal estimates with a 1.1 µm (95% CI 0.6–1.6 µm) increase per 3 µg/m^3^. Living near a major roadway was associated with decreased rather than increased venular diameter, but this association was not statistically significant in the fully adjusted model (results not shown). Short-term exposures were not associated with venular diameters (Table 3).

## Discussion

In a large population-based cohort study of adults without preexisting cardiovascular disease, we found independent associations between long- and short-term concentrations of fine particulate air pollution and vessel diameters of the retinal microvasculature, as measured using standardized photographic methods. Increased air pollution concentrations were associated with retinal arteriolar narrowing, an outcome that has previously been associated with increased risk of myocardial infarction, stroke, hypertension, and cardiovascular mortality, independent of other traditional risk factors. [13]–[19].

An increase of 3 µg/m^3^ in long-term PM_2.5_ concentrations predicted at participant homes was associated with a −0.8 µm or −0.6% decrease in arteriolar diameter. This decrease is of the same order of magnitude as that of more traditional risk factors, with reductions equivalent to those seen for a 7-y increase in age or a 3-mm Hg increase in diastolic blood pressure in this cohort. Daily increases in PM_2.5_ concentrations were similarly associated with narrower arteriolar diameters, though these associations were not statistically significant (*p* = 0.09). All associations persisted after control for hypertension and other factors previously reported to be associated with narrower retinal arterioles and venular diameters. Although positive associations were demonstrated between chronic exposures to ambient PM_2.5_ and venular diameters, increases that have been associated with risk of stroke [13],[19], these relationships were less robust and less consistent across exposure metrics than those found for CRAE.

This investigation is, to our knowledge, the first study to directly examine the relationship of ambient air pollution and in vivo measures of the human microvasculature. This study adds to previous in vivo work on the impacts of air pollution focused on larger vessels and extends past investigations on the ocular manifestations of environmental exposures [37]–[40] by evaluating them in a population-based cohort with high quality information on exposure, outcome, and confounders. Our findings support the hypothesis that subclinical microvascular changes are associated with PM exposures, even within the range of concentrations common in many developed countries with adopted pollution control measures and at levels much lower than are often found in newly industrialized urban centers. Finally, jointly modeling the short- and long-term impacts of a pollutant on vascular health provides new evidence that both time frames may be relevant for this outcome and enlarges the limited knowledge base in an area of substantial scientific uncertainty.

Our results are generally consistent with the existing literature on air pollution and the human vasculature, which has thus far examined associations with vessel diameter and function in the forearm. Several studies have used ultrasound to study the brachial artery, a conduit vessel with distinct anatomical and functional properties. In experimental settings, short-term exposures to high levels of air pollutants have been shown to elicit vasoconstriction of the brachial artery but not impair endothelium-dependent dilation [12],[41]–[43]. In observational studies, short-term exposures have been associated with impaired endothelium-dependent vasodilation [9],[11]. Though the brachial artery assesses endothelial function in a vessel comparable to the epicardial coronary arteries, it has not been shown, to date, to be generalizable to the microvasculature of the heart and lungs, which are critically important in the progression of clinical vascular disease [44].

Other studies, which have examined an amalgamation of vessels smaller than the brachial artery using forearm plethysmography have similarly shown diminution in forearm blood flow following short-term exposure to diesel exhaust [10],[45]. Furthermore, recent toxicological investigations have demonstrated that acute exposures to PM blunt arteriolar dilatation of skeletal and subepicardial muscle in rats. These studies also demonstrated increased leukocyte adhesion and rolling in paired venules, elevated leukocyte deposition, increased oxidative stress, and decreased bioavailability of nitric oxide [7],[44],[46],[47]. Research also has suggested that air pollution can contribute to elevated blood pressure in humans [43],[48].

Our findings of widened venular diameters with chronic exposures to air pollution are similarly consistent with the literature based on investigations of cigarette smoke [23],[49]. Although the biological mechanisms for these associations remain to be fully elucidated, it is thought that CRVE widening might be, at least partially, due to systemic inflammation [23]. Nevertheless, our observed associations for CRVE should be interpreted with caution as they were not robust to exposure parameterization or timing of exposure, were driven entirely by differences between the two cities with the highest and lowest ambient air pollution concentrations, and were sensitive to control for CRAE.

Taken together, our findings support an increasingly consistent view of the downstream mechanisms through which particulate matter exposures might cause the clinical cardiovascular disease observed to be associated with air pollution in epidemiological studies. As such, these results further enhance the biological plausibility of observed air pollution–disease relationships. Although we cannot directly identify the upstream pathways of short and long-term exposures to PM_2.5_ that might have caused the observed narrowing of retinal arterioles, the fact that our findings persisted following adjustment for blood pressure and hypertension status may imply that these microvasculature changes are not a response to other changes in vascular resistance.

Our findings of significantly narrower retinal arteriolar diameters (and significant widening of retinal venular diameters) among persons residing in areas with higher long-term concentrations of air pollution are unique and support many published findings of elevated cardiovascular mortality risk in areas of elevated pollution. [50]–[52]. The observation of independent associations with microvascular phenomenon for short-term and long-term concentrations of PM_2.5_ is consistent with an impact of chronic pollution exposure that exceeds what can be ascribed to short-term increases in pollutant concentrations. Although we cannot fully exclude the possibility of confounding between short- and long-term associations without temporally resolved spatial predictions on a shorter time scale, this joint modeling method may help to further disentangle acute and long-term relationships with the cardiovascular system.

It should be noted that our long-term exposure findings were driven by between-city differences in pollution concentrations, most likely as the result of enhanced variability in PM_2.5_ concentrations between cities as compared to within any city. Our findings, however, are supported by a nonsignificant negative association with our pooled estimate of within-city variability, a lack of sensitivity of the arteriolar results to exclusion of any one particular city, and consistent negative associations for short-term fluctuations in pollution that are independent of city. The observed associations are also unlikely to be due to differences in data collection as standardized methods were used to maintain consistency across sites in retinal image collection. Similarly, all measures were read in a masked manner by a single centralized reading center using a validated protocol to standardize the grading of image parameters. Finally, our results also were found to be qualitatively similar across models with differing levels of control for many potential confounders (individual-level and city-wide).

Another point to note is that there was a relatively high proportion of missing data (16%) as a result of incomplete participant geocoding. Although individuals in rural or newer areas might be less likely to be geocoded, we do not anticipate any bias to our analysis since the likelihood of data being missing should be unrelated to the outcome.

Overall, these findings may have practical relevance since relationships with subclinical microvascular changes were found at PM_2.5_ levels that commonly occur in developed nations and well below those in developing countries. With a mean long-term concentration of 16 µg/m^3^, these levels were similar to the annual National Ambient Air Quality Standard of 15 µg/m^3^ established by the US EPA for PM_2.5_. The observation of a linear trend in the relationship between PM_2.5_ and CRAE further supports past findings [6], which have failed to find evidence of a threshold and suggest that the cardiovascular effects of air pollution can occur at levels below existing regulatory standards. Since different airborne pollutants can be correlated in the environment, we cannot exclude the possibility that these results might also reflect the toxicity of another correlated pollutant or the pollution mixture as a whole. As the MESA Air project proceeds with additional refinements to the air pollution estimates, including indoor predictions and investigation of other copollutants, and the MESA study collects an additional round of retinal photography in this cohort, we will be able to confirm our findings and examine this phenomenon in greater detail.

### Conclusion

Long- and short-term concentrations of fine particulate matter were associated with observable differences in microvascular structure in a cross-sectional analysis of a large cohort, characterized by high quality information on exposure, outcome, and potential confounders. Individuals with higher long- and short-term air pollution concentrations had narrower arteriolar diameters, even after control for typical risk factors. We also found associations between chronic but not acute air pollution levels and widened retinal venules, another marker of microvascular disease, although these associations were less robust. Overall, our findings enhance the biological plausibility of the hypothesis that important vascular phenomena are associated with small increases in short-term or long-term air pollution exposures, even at current exposure levels. These results further support reported associations between air pollution and the development and exacerbation of clinical cardiovascular disease.

## Abbreviations

AQS: Air Quality System
BMI: body mass index
CI: confidence interval
CRAE: central retinal arteriolar equivalents
CRVE: central retinal venular equivalents
df: degrees of freedom
EPA: Environmental Protection Agency
HDL: high-density lipoprotein
LDL: low-density lipoprotein
MESA: Multi-Ethnic Study of Atherosclerosis
MESA Air: Multi-Ethnic Study of Atherosclerosis and Air Pollution
PM_2.5_: fine particulate matter less than 2.5 µm in aerodynamic diameter
SD: standard deviation
